# ID 247 - Falar de ATS é Falar sobre Histórias de Vida: o papel de uma associação de pacientes na educação e qualificação da participação social em incorporações de tecnologias para fibrose cística

**DOI:** 10.5327/2237-9622.2025.v34s1.247

**Published:** 2025-11-25

**Authors:** Verônica Stasiak Bednarczuk de Oliveira, Vinícius Bednarczuk de Oliveira, Marise Basso Amaral, Marilis Dalarmi Miguel

## Abstract

**Introdução:**

Associações de pacientes desempenham um papel importante de educação em saúde, especialmente nos processos de Avaliação de Tecnologias em Saúde (ATS). Segundo Oliveira (2024), 57% dos participantes das Consultas Públicas (CPs) sobre medicamentos para fibrose cística (FC), realizadas em 2020 e 2022, souberam da abertura das CPs por meio de associações de pacientes. Isso evidencia o papel dessas organizações na disseminação de informações e na capacitação da comunidade para participar de maneira qualificada nos processos de ATS.

Este trabalho descreve iniciativas do Instituto Unidos pela Vida (IUPV) no fortalecimento da comunidade de FC, promovendo educação em ATS e participação social, resultando em contribuições qualificadas com evidências de vida real para o processo de incorporação de moduladores no SUS.

Para educar a comunidade e contribuir pela qualificação da jornada de engajamento, o IUPV estruturou suas atividades em três etapas principais, realizadas a partir da aprovação do primeiro modulador em 2012. Exemplos:

1. Pré-submissão à Conitec: a partir da aprovação do primeiro modulador pelo FDA em 2012, o IUPV produziu centenas de vídeos, textos, podcasts e eventos educativos sobre a fibrose cística, novos tratamentos e medicamentos, e sua jornada no sistema de saúde, desde a descoberta até a dispensação aos pacientes. Seis encontros anuais nacionais foram realizados com líderes de associações de FC para capacitação em ATS e políticas públicas, além de ações de e controle social.
2. Pós-submissão à Conitec: ações com foco no monitoramento de prazos e processos, fortalecimento da perspectiva do paciente e promoção de debates em congressos e eventos, como três edições do Simpósio Brasileiro de FC, duas edições do Fórum Brasileiro de ATS para Doenças Raras, além de mesa estratégica no Congresso Brasileiro de FC. Também foram realizadas publicações científicas sobre evidências de vida real e pesquisas acadêmicas que analisaram e evidenciaram problemas em consultas públicas anteriores, de tecnologias para FC.
3. Pré e pós-consulta pública: a preparação da comunidade para as CPs incluiu a produção de materiais informativos, vídeos, podcasts, materiais com passo a passo, suporte diário e publicações em mídias sociais, impactando mais de 61 mil pessoas somente na CP do modulador incorporado em 2024. O IUPV gerou contribuições técnicas e de experiência e realiza controle social após a CP até a dispensação dos medicamentos no SUS.

Além dessas etapas, o IUPV realizou projetos como o Caderno Especial sobre Participação Social no SUS, além do projeto de pesquisa Qualifica SUS, que mapeou o conhecimento das associações sobre ATS e participação social. Também desenvolveu a plataforma gratuita Conexão SUS, com cursos sobre ATS e políticas públicas, para capacitar a comunidade. A incorporação de dois moduladores no SUS resultou não apenas da qualidade da participação social, mas da robustez de evidências clínicas e da redução do impacto orçamentário. A experiência do IUPV demonstra que a educação em ATS pode qualificar a participação social no SUS, destacando, por fim, a necessidade de que os decisores considerem, de fato, as evidências de vida real obtidas por meio desses mecanismos.

Essas iniciativas serão apresentadas no eixo Democratizando a ATS, no Congresso da Rede Brasileira de Avaliação de Tecnologias em Saúde (Rebrats), através de apresentação em PowerPoint com resultados sistematizados, fluxograma e imagens.

**Método:**

Monitor para apresentação de slides. Não é necessário equipamento de som.

